# Supplementary material for: Agreement between smartphone-based mobile sensing and actigraphy sleep metrics in young people with bipolar disorder
Source: medRxiv. 2026 Mar 2:2026.02.20.26346722. Preprint. [Version 1] doi: 10.64898/2026.02.20.26346722 (PMC13004090; doi:10.64898/2026.02.20.26346722)
Supplement: Supplement 1 [file NIHPP2026.02.20.26346722v1-supplement-1.pdf]

medRxiv preprint doi: https://doi.org/10.64898/2026.02.20.26346722; this version posted March 2, 2026. The copyright holder for this preprint (which was not certified by peer review) is the author/funder, who has granted medRxiv a license to display the preprint in perpetuity.
It is made available under a CC-BY 4.0 International license .

eSupplement

eTable 1. Comparing Included vs. Excluded Sample on Demographic and Clinical Characteristics

Variable	Actigraphy Sample (n=23)	Excluded Sample (n=30)	Test Statistic	p-value
Intake Age, mean (standard deviation)	21.3 (2.4)	22.3 (2.1)	Z=1.46	0.1435
Intake Diagnosis				
Bipolar Disorder-I, %	78.3%	73.3%	FET	0.7558
Bipolar Disorder-II, %	21.7%	26.7%		
Female, %	82.6%	93.3%	FET	0.3848
Race				
Black, %	4.4%	0.0%		
Asian, %	13.0%	0.0%	FET	**0.0039**
White, %	60.9%	96.7%		
Multiracial, %	21.7%	3.3%		
Socioeconomic Status, mean (standard deviation)	4.1 (0.9)	3.6 (1.1)	Z=1.72	0.0861
Global Assessment of Functioning, mean (standard deviation)	71.7 (9.0)	76.0 (7.6)	Z=1.81	0.0708
Generalized Anxiety Disorder, %	52.2%	73.3%	FET	0.1515
Attention Deficit Hyperactivity Disorder, %	21.7%	36.7%	FET	0.3661
Psychosis, %	4.4%	0.0%	FET	0.4340
Substance Use Disorder, %	34.8%	36.7%	FET	~1
Family History of Bipolar Disorder, %	30.4%	20.0%	FET	0.5217

FET = Fisher's Exact Test

medRxiv preprint doi: https://doi.org/10.64898/2026.02.20.26346722; this version posted March 2, 2026. The copyright holder for this preprint (which was not certified by peer review) is the author/funder, who has granted medRxiv a license to display the preprint in perpetuity.
It is made available under a CC-BY 4.0 International license .

eTable 2. Phone Use Questionnaire

**Where do you keep your smartphone at night?**	**n**	**%**
In Bed	13	56.5
Within reach of bed	9	39.1
In room, but out of reach	1	4.4
In another room	0	0.0
**When you wake up in the middle of the night, how often do you check your smartphone?**	**n**	**%**
Pretty much every time	6	26.1
Most of the time	3	13.0
Some of the time	9	39.1
Almost never	3	13.0
Not Applicable: WASO Rare	2	8.7
**How many minutes before bedtime do you usually stop using your smartphone?**	**n**	**%**
Less than 5 minutes	16	69.6
Less than 30 minutes	7	30.4
More than 30 minutes	0	0.0
**After waking up, when do you first check your smartphone?**	**n**	**%**
Within 5 minutes or use as alarm	23	100.0
Within 30 minutes	0	0.0
More than 30 minutes later	0	0.0
**While you are awake, how often do you keep your smartphone within reach?**	**n**	**%**
Pretty much all the time	17	73.9
Most of the time	6	26.1
Less than half of the time	0	0.0
Almost never	0	0.0
**While you are awake, and not at school or work, how often do you check your smartphone?**	**n**	**%**
A few times an hour	22	95.7
Hourly	1	4.4
3-4 times a day	0	0.0
Once daily or less	0	0.0
**While you are at school or work, how often do you check your smartphone?**	**n**	**%**
A few times an hour	14	60.9
Hourly	6	26.1
Once in a while	3	13.0

medRxiv preprint doi: https://doi.org/10.64898/2026.02.20.26346722; this version posted March 2, 2026. The copyright holder for this preprint (which was not certified by peer review) is the author/funder, who has granted medRxiv a license to display the preprint in perpetuity.
It is made available under a CC-BY 4.0 International license .

eTable 3. Sensitivity Analysis: Complete Mobile Sensing Days Only

Sleep Variable	AWARE Means	Actigraphy Means	RMSE	Subject-Aggregated RMSE	β	95% CI		p-value
Sleep Onset	11:51 PM	12:18 AM	19.4	30.6	0.66	0.59	0.73	<0.0001
Midsleep	3:48 AM	4:36 AM	12.9	18.7	0.76	0.69	0.82	<0.0001
Sleep Offset	7:51 AM	8:54 AM	18.4	24.3	0.69	0.62	0.76	<0.0001
Total Sleep Time	8.11 hours	7.81 hours	20.8	26.5	0.52	0.44	0.60	<0.0001

RMSE = Root Mean Squared Error in minutes, β = Linear Mixed Regression Coefficient (Standardized), CI = Confidence Interval

eTable 4. Sensitivity Analysis: participants who reported they stopped using their smartphones less than five minutes before bedtime (n=16)

Sleep Variable	RMSE	Subject-Aggregated RMSE	β	95% CI		p-value
Sleep Onset	25.5	31.2	0.65	0.56	0.74	<0.0001
Midsleep	16.4	18.1	0.74	0.66	0.81	<0.0001
Total Sleep Time	24.4	24.1	0.57	0.47	0.66	<0.0001

RMSE = Root Mean Squared Error in minutes, β = Linear Mixed Regression Coefficient (Standardized), CI = Confidence Interval

eTable 5. Expanding mobile sensing sleep detection interval to 7 p.m. - 1 p.m.

Sleep Variable	RMSE	Subject-Aggregated RMSE	β	95% CI		p-value
Sleep Onset	61.5	78.6	0.16	0.08	0.23	<0.0001
Midsleep	24.7	30.0	0.35	0.25	0.45	<0.0001
Sleep Offset	23.9	28.5	0.44	0.34	0.53	<0.0001
Total Sleep Time	65.9	76.4	0.16	0.06	0.26	0.0024

RMSE = Root Mean Squared Error in minutes, β = Linear Mixed Regression Coefficient (Standardized), CI = Confidence Interval

medRxiv preprint doi: https://doi.org/10.64898/2026.02.20.26346722; this version posted March 2, 2026. The copyright holder for this preprint (which was not certified by peer review) is the author/funder, who has granted medRxiv a license to display the preprint in perpetuity.
It is made available under a CC-BY 4.0 International license .

eTable 6. Sensitivity analyses by smartphone operating system

Sleep Variable	Operating System	RMSE	Subject-Aggregated RMSE
Sleep Onset	Android (n=5)	42.5	58.5
Midsleep		22.5	30.2
Sleep Offset		20.5	29.2
Total Sleep Time		38.3	39.7
Wake after Sleep Onset		51.9	54.8
Sleep Onset	iOS (n=18)	18.1	19.9
Midsleep		14.2	15.1
Sleep Offset		20.2	22.2
Total Sleep Time		18.3	18.4
Wake after Sleep Onset		48.6	44.4

RMSE = Root Mean Squared Error in minutes

eTable 7. Exploratory analysis of weekend/weekday moderation effects

Sleep Variable	Weekend/Weekday	β	95% CI		p-value	Type 3 Interaction p-value
Sleep Onset	Weekend	0.79	0.66	0.91	<0.0001	0.0229
	Weekday	0.62	0.53	0.72	<0.0001	
Midsleep	Weekend	0.83	0.72	0.94	<0.0001	0.0399
	Weekday	0.70	0.62	0.78	<0.0001	
Sleep Offset	Weekend	0.79	0.66	0.92	<0.0001	0.0324
	Weekday	0.63	0.54	0.71	<0.0001	
Total Sleep Time	Weekend	0.67	0.53	0.81	<0.0001	0.0162
	Weekday	0.48	0.38	0.57	<0.0001	

β = Linear Mixed Regression Coefficient (Standardized), CI = Confidence Interval

medRxiv preprint doi: https://doi.org/10.64898/2026.02.20.26346722; this version posted March 2, 2026. The copyright holder for this preprint (which was not certified by peer review) is the author/funder, who has granted medRxiv a license to display the preprint in perpetuity.
It is made available under a CC-BY 4.0 International license .

eTable 8. Exploratory analysis of weekend/weekday moderation effects

Sleep Variable	Moderator	β	95% CI		p-value	Type 3 Interaction p-value
Sleep Onset	Concurrent Mood Symptoms*	0.76	0.63	0.89	<0.0001	0.2215
	No Mood Symptoms	0.67	0.58	0.75	<0.0001	
Midsleep	Concurrent Mood Symptoms*	0.89	0.78	1.00	<0.0001	0.0026
	No Mood Symptoms	0.68	0.59	0.76	<0.0001	
Sleep Offset	Concurrent Mood Symptoms*	0.83	0.70	0.97	<0.0001	0.0052
	No Mood Symptoms	0.59	0.49	0.69	<0.0001	
Total Sleep Time	Concurrent Mood Symptoms*	0.54	0.39	0.70	<0.0001	0.9535
	No Mood Symptoms	0.54	0.43	0.64	<0.0001	
Sleep Onset	GAD	0.71	0.62	0.80	<0.0001	0.0330
	No GAD	0.54	0.41	0.67	<0.0001	
Midsleep	GAD	0.80	0.72	0.88	<0.0001	0.0205
	No GAD	0.60	0.46	0.75	<0.0001	
Sleep Offset	GAD	0.68	0.59	0.77	<0.0001	0.8396
	No GAD	0.70	0.56	0.84	<0.0001	
Total Sleep Time	GAD	0.52	0.41	0.62	<0.0001	0.3592
	No GAD	0.60	0.45	0.75	<0.0001	

β = Linear Mixed Regression Coefficient (Standardized), CI = Confidence Interval

medRxiv preprint doi: https://doi.org/10.64898/2026.02.20.26346722; this version posted March 2, 2026. The copyright holder for this preprint (which was not certified by peer review) is the author/funder, who has granted medRxiv a license to display the preprint in perpetuity.
It is made available under a CC-BY 4.0 International license .

eFigure 1. Expanding mobile sensing sleep detection interval to 7 p.m. - 1 p.m.

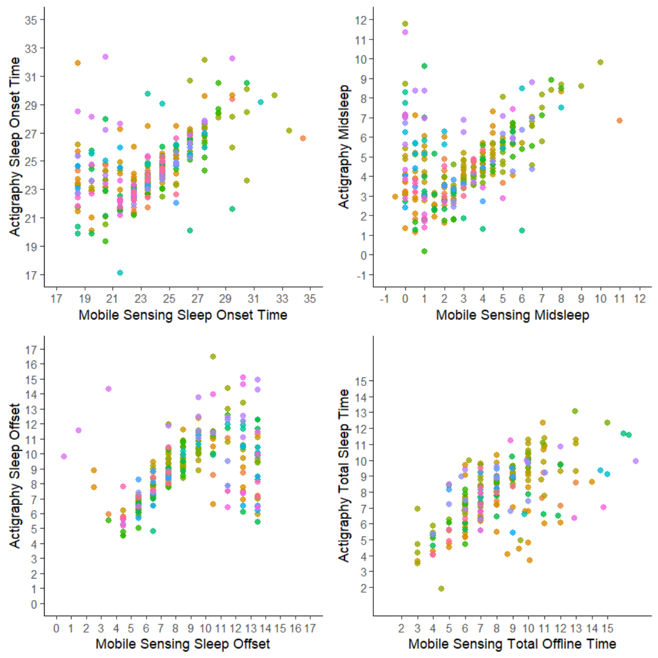

Points of the same color reflect repeated observations within-participant.
